# Severe obesity increases more rapidly in Brazil than moderate obesity: analysis of Vigitel 2006–2021

**DOI:** 10.1590/1980-549720250011

**Published:** 2025-03-21

**Authors:** Marcos Brum, Roland Sturm

**Affiliations:** I Pontifícia Universidade Católica do Rio Grande do Sul – Porto Alegre (RS), Brazil.; II RAND, Santa Monica, California, United States.

**Keywords:** Morbid obesity, Prevalence, Public health, Epidemiology, Obesidade mórbida, Prevalência, Saúde pública, Epidemiologia

## Abstract

This study analyzes how the rates of increase in Brazil differ by obesity class. The paper estimates time trends for extreme weight categories from 2006 to 2021 and extrapolates through 2025 using Vigitel. Comparing 2021 to 2006 rates, the prevalence of BMI≥45 increased by 152%, BMI≥40 by 120%, and BMI≥35 by 104%. In contrast, BMI≥30 increased by 66%. Results are adjusted for demographic changes. Severe obesity is increasing far more rapidly than what commonly reported obesity statistics indicate. These groups have higher health burdens and healthcare needs, and the health system needs to be prepared to see such individuals regularly.

## RESUMO

Este estudo analisa como as taxas de aumento no Brasil diferem por classe de obesidade. O trabalho estima tendências temporais para categorias de peso extremo de 2006 a 2021 e faz uma projeção até 2025 usando dados do Vigitel. Comparando 2021 a 2006, a prevalência de IMC≥45 aumentou 152%, IMC≥40 subiu 120% e IMC≥35 subiu 104%. Em contraste, o IMC≥30 aumentou 66%. Os resultados foram ajustados para mudanças demográficas. A obesidade grave está crescendo muito mais rápido do que os dados usuais sobre obesidade indicam. Esses grupos apresentam maiores demandas de saúde, e o sistema de saúde precisa se preparar para atender essas pessoas regularmente.

## INTRODUCTION

Between 2006 and 2016, the average body mass index (BMI) of adult Brazilians has increased by 1 BMI unit, resulting in an increase in obesity from 12% to 18% based on data from Vigitel^1,2^. This trend has continued, and the Ministério da Saúde reported an obesity prevalence of 24.3% in 2023^3^.

Defining obesity by a BMI of over 30 obscures the heterogeneity among that group. Severe obesity (e.g., BMI over 40) entails complications and challenges different from moderate obesity (e.g., BMI between 30 and 35). If the etiology of severe obesity differs from moderate obesity, there is no reason to expect similar trends. For example, if morbid obesity were caused by genetic disorders, its prevalence would not be affected by general population changes in diet and behaviors. The objective of this paper is to compare how (and whether) rates of increase by obesity class vary in Brazil.

Surveys generally lack the sample size to identify trends for severe obesity descriptively. Descriptive statistics for different categories of obesity by state and year have been reported for Brazil, but confidence intervals are so large that there are no insights into differential trends^1,2^. The solution in other countries has been to estimate trends by smoothing random fluctuations and adjusting for changes in the composition of samples using regression. Using a survey similar to the Brazilian Vigitel and regression methods, the prevalence of severe obesity was found to grow at a much faster rate than that of moderate obesity^4^. Similar trends were also reported in Spain and Sweden^5,6^.

## METHODS

### Study design

This study is a population-based repeated cross-sectional analysis using data from Vigitel from 2006 to 2021.

### Data source

Vigitel is a monitoring tool of the Ministry of Health to track health behaviors and risk factors across Brazil. Vigitel is conducted annually in 26 state capitals and the Federal District, with an approximate sample of 2,000 individuals per city each year, and includes weights that adjust for the unequal probability of selecting households and differential response rates. Being a telephone survey, all data collected reflects respondent self-report. This is a limitation because self-reported height and weight tend to systematically vary from objective measurement. While less likely to affect the trend estimates reported here (biases would be similar across years), it affects the absolute magnitudes. A second limitation is that Vigitel was limited to state capitals until 2021. The data used in this study are publicly available and can be accessed freely at [https://svs.aids.gov.br/download/Vigitel/].

Between 2006 and 2021, Vigitel recorded a total of 784,479 records. To reduce data errors in reporting and data entry, we drop unlikely or implausible values: age over 100 years, height below 100 cm or above 213 cm, weight below 45 kg or above 227 kg. The analysis distinguishes the following (overlapping) categories: BMI≥30, BMI≥35, BMI≥40, and BMI≥45.

We exclude pregnant women and participants with missing data from the analysis. This led to the exclusion of 129,864 records, resulting in an analysis sample of 654,615 records. The study period ends in 2021 because Vigitel was not fielded in 2022 and in 2023 used a different sampling design.

### Statistical analysis

The statistical analysis of this study was performed using individual-level logistic regression, with different obesity categories as the dependent variable (a separate equation for each category). Time trends are modeled using linear spline functions, with knots set in 2012 and 2018. This provides a flexible representation of time trends, with different trends across each 6-year period. Splines smooth the estimates in comparison to year indicators, especially as sample sizes decreased in recent years. Additional regressor variables include sociodemographic factors to separate the trend in weight gain from changes in population demographics such as age (grouped as 18–30, 31–49, 50–59, 60–69, and 70+), education level (categorized as 0–8, 9–11, and 12 or more years), race/color (white, black, yellow, brown, and indigenous), and sex. To account for the clustering in Vigitel’s sampling design by state, indicator variables for state capitals were included in the model. Predictions are weighted to be representative of the Brazilian population, using the peso_rake variable.

Results are presented as percentage increases relative to 2006. The model also extrapolates trends through 2022–2025. The adjusted results reflect the sociodemographic composition of the 2021 survey. The data were analyzed using the R programming language (RStudio version 4.4.2.).

## RESULTS

**Figure 1:**
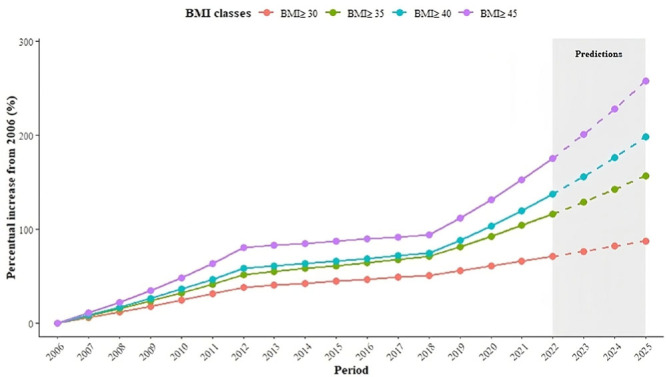
Growth of prevalence by severity of obesity. Body Mass Index indicates body mass index (calculated as weight in kilograms divided by the square of height in meters).

Figure 1 presents the percent increase for different BMI categories in Brazil relative to their 2006 values. During this period, the prevalence of BMI≥45 increased by 152% in 2021 over 2006 values. In contrast, obesity defined as a BMI≥30 increased by 87%. The prevalence of BMI≥45 is expected to be 258% higher in 2025 than in 2006.

The results shown are adjusted for changes in population characteristics. However, neither population aging nor increasing numbers of minority groups play a major role in obesity trends, and results would be very similar without the demographic adjustment (results not shown).

## DISCUSSION

The findings of this study highlight an underappreciated problem: Severe obesity is increasing at a much faster rate than moderate obesity. The results for Brazil parallel previously reported trends in the United States, Sweden, and Spain^4-6^.

The implications for public health are significant. Individuals with a BMI≥40 or BMI≥45 are at far higher risk of developing serious comorbidities, especially type 2 diabetes and cardiovascular diseases. At the same time, they encounter more difficulties in accessing health care due to functional limitations and, at times, the lack of equipment for their body size in medical offices.

Because the prevalence of severe obesity is increasing much faster than that of moderate obesity, average estimates of obesity effects obscure real consequences for individuals, physician practices, hospitals, SUS, and private health plans. The findings highlight an additional and possibly unexpected burden on the Brazilian healthcare system in recent years and the foreseeable future.

## References

[1] Malta DC, Silva AG, Tonaco LAB, Freitas MIF, Velasquez-Melendez G. (2019). Time trends in morbid obesity prevalence in the Brazilian adult population from 2006 to 2017.. Cad Saude Publica.

[2] Flores-Ortiz R, Malta DC, Velasquez-Melendez G. (2019). Adult body weight trends in 27 urban populations of Brazil from 2006 to 2016: a population-based study.. PLoS One.

[3] Brasil. (2024). Ministério da Saúde. Dia Mundial da Obesidade: conscientização e desafios no combate a uma epidemia global [Internet]..

[4] Sturm R, Hattori A (2013). Morbid obesity rates continue to rise rapidly in the United States.. Int J Obes (Lond).

[5] Basterra-Gortari FJ, Bes-Rastrollo M, Ruiz-Canela M, Gea A, Martinez-Gonzalez MA (2017). Prevalence of obesity and diabetes in Spanish adults 1987–2012.. Med Clin (Barc).

[6] Neovius M, Teixeira-Pinto A, Rasmussen F (2008). Shift in the composition of obesity in young adult men in Sweden over a third of a century.. Int J Obes (Lond).

